# Characterizing the intrinsic parameters, mass, volume and density, of single pollen grains as a base for mechanical analysis of pollen adhesion

**DOI:** 10.1007/s00425-026-05111-x

**Published:** 2026-08-07

**Authors:** Martin Becker, Stanislav Gorb

**Affiliations:** Department of Functional Morphology and Biomechanics, Zoological Institute, Kiel University, Am Botanischen Garten 1–9, 24118 Kiel, Germany

**Keywords:** Adhesion, Density, Microscopy, Pollen, Pollination, Weight measurements

## Abstract

**Main conclusion:**

This study combines own experimental data with an extensive literature research to characterize the average single pollen grain mass, volume and density as parameters for future force measurements on pollen.

**Abstract:**

The analysis of pollen grain mechanical properties and adhesion and detachment forces is important to understand the process of pollination in general. However, it requires knowledge of pollen grain mass, volume and density. Despite their importance for research, mass and density have rarely been measured directly, but there is vast data about pollen grain size and shape. Thus, an average value of pollen grain density can provide an estimation of single grain mass for a given volume. However, in this study, we directly measured mass and volume of pollen grains from five anemophilous species by using the weigh-and-count approach and the projected-particle-area approach. This data was used to calculate the density for fresh and dry grains. Furthermore, we combined these results with an extensive literature research to provide a range for the average density of pollen from eudicots, monocots and gymnosperms. We found an uncertainty of up to ± 30% for density calculation, as well as some significant differences between the three plant groups. Methodical details and side effects that can influence pollen grain parameters are discussed. This study is an attempt to extend existing geometrical data about pollen size with the physical parameters, such as mass and density, to provide a background for further biomechanical analysis of pollen grains and pollination process.

**Supplementary Information:**

The online version contains supplementary material available at 10.1007/s00425-026-05111-x.

## Introduction

Pollination is the most critical step of reproduction in higher plants relying on the transfer of the pollen grain from the stamen of one plant individual to the stigma of another one. This transfer is provided by an external vector, which can be insects or other animals in the case of zoophilous species or abiotic vectors like in wind-pollinated (anemophilous) species (Willmer 2011). At each stage of this process, the pollen has to be kept in place until the conditions are optimal for transfer to the next place; therefore, the interplay of adhesion and detachment forces, acting on a pollen grain, is important to understand this process. However, adhesion depends on various factors, including the general shape of a pollen grain and its orientation, as well as the surface structure and the presence/absence of coating layers (pollenkitt). In addition, the properties of the substrate, to which the grain adheres, affect the contact conditions and therefore the adhesion force. Thus, predicting pollen grain adhesion based on these properties is at least difficult if not impossible and so far, only a few studies measured adhesion forces of pollen directly, using atomic force microscopy (AFM) (Thio et al. 2009; Lin et al. 2013; Qu 2017; Ito and Gorb 2019; Huth et al. 2022). These studies showed strong differences in adhesion, depending on the species and individual contact conditions, making a comparable analysis very difficult.

Another approach is the analysis of detachment forces, as in the case of a detachment event, these forces are at least equal to the current adhesion and can therefore be used as an approximation. In nature, the majority of anemophilous species relies on passive detachment by either aerodynamic forces or vibration forces. In case of the former, the critical wind speed for release can be calculated, depending on the pollen grain´s size and density as well as on its adhesive properties (Timerman and Barrett 2021). On the other hand, vibration of stamen or inflorescences can be translated to a centrifugal acceleration acting on the pollen grain and causing detachment, and this depends on the grain mass (King and Buchmann 1996). The latter case can also be simulated experimentally with a laboratory centrifuge, as described in our previous study (Becker and Gorb 2025). Also, many other studies used centrifugation (Luu et al. 1997; Lin et al. 2016; Huth et al. 2022) or vibration (Urzay et al. 2009; Timerman 2013) for analysis of pollen grains. However, each of the mentioned approaches to characterize pollen detachment requires knowledge of the pollen grains’ intrinsic parameters, such as mass (***m***), density (***ρ***) and volume (***V***), which are related to each other:

$$m \, = \, \rho \, * \, V.$$

This approach enables also comparative analysis of adhesion properties in various species. Nevertheless, these intrinsic parameters of microparticles are difficult to measure and, in the case of pollen, they can also vary over time due to changes in water content (Pacini 2000).

Previous studies have described several methods to analyze these parameters. Pollen grain volume can be estimated from microscopic images, by measuring one or more characteristic diameters and calculating the equivalent spherical volume. The majority of previous studies used light microscopy (LM) images (Payne 1972; Nepi et al. 2001; Sosnoskie et al. 2009), while scanning electron microscopy (SEM) images were used less often (Niklas 1988; Pope 2010). However, for irregular shaped or crumpled pollen, it can be difficult to measure the correct diameters. In this case, the projected particle area (PPA) provides an alternative way to calculate the equivalent spherical volume (Van Hout and Katz 2004; Ribeiro et al. 2015; Galveias et al. 2022). Weight measurements in some cases only considered the total mass of a huge sample of grains, as the aim was to measure relative changes due to hydration (Nepi et al. 2001) or loss of pollenkitt (Gilissen and Brantjes 1978). On the other hand, calculation of the mass of a single grain requires counting of the accurate number of grains in the sample, which can be done, for example, using a hemocytometer (Bunderson and Levetin 2015). Other methods use the electrodynamic balance (EDB) (Laucks et al. 2000; Griffiths et al. 2012) or a cantilever-based resonator (Chan et al. 2014), both of which can be used to directly measure the mass of a single pollen grain. Finally, values for pollen grain density were provided by many studies that measured the settling velocity of single grains in still air and calculated the density using Stokes´s law (Jackson and Lyford 1999; Hirose and Osada 2016; Klahs 2022). Nevertheless, such information about pollen grain mass or density is still rather rare.

On the other hand, a huge proportion of land plants has been investigated in terms of their pollen morphology (Blackmore 2007), and platforms like PalDat (paldat.org) provide detailed information about their shape and size. While the calculation of volume still might be difficult in some cases, an approximate value of pollen grain density can provide a calculation of the single grain mass for a given volume and the other way round. Furthermore, this information can then be used to simulate the critical conditions for pollen grain detachment under various experimental or natural conditions and compare it to measurement results. Finally, comparison of theoretically expected and truly measured detachment forces enables characterization of pollen adhesion on different surfaces that can again be used as a basis for hypotheses about functional morphology and adaptation of pollination mechanisms. Therefore, the characterization of pollen grain density plays a key role in this approach.

In this study, we provide extensive calculation of single pollen grain mass and volume of five anemophilous species in two states, “fresh” and “aged” and used this data to calculate their density. We used cryo-SEM, to depict pollen in fresh state for volume calculation. Furthermore, we combined these results with data from our previous centrifugation experiment (Becker and Gorb 2025) to provide the distribution of measured pollen grain adhesion forces for each species. We have chosen the following plant species: the gymnosperm representative scots pine (*Pinus sylvestris* Linné) (Pinaceae), a grass species—perennial ryegrass (*Lolium perenne* Linné) (Poaceae) and hazelnut (*Corylus avellana* Linné) (Betulaceae). Furthermore, we chose a special case within the usually insect-pollinated Asteraceae family—mugwort (*Artemisia vulgaris* Linné) (Asteraceae) and ribwort plantain (*Plantago lanceolata*, Linné) (Plantaginaceae) as a member of the ambophilous (using both, biotic and abiotic pollination vectors) genus *Plantago*. Finally, we combined the measured values for mass and volume with literature data about more than 70 species and made a regression analysis to provide a value for the average density of pollen grains from the three large groups of land plants: eudicots, monocots and gymnosperms. This study provides an attempt to amplify the enormous database of pollen grain morphology with further up-to-date information about single grain mass and density to enable complete characterization of pollen grain properties and their biomechanics during pollination in future studies.

## Materials and methods

### Specimens and previous methods

Pollen grains of *Artemisia vulgaris, Corylus avellana, Plantago lanceolata, Pinus sylvestris* and *Lolium perenne* were collected from individual plants, during their flowering period, in the Botanical Garden of Christian-Albrechts-University Kiel, Federal country Schleswig–Holstein, Northern Germany. Samples of *P. sylvestris* were all taken from the same tree in a half shadow location. Samples of *C. avellana* were taken from three shrubs at the same spot also in a half shadow location. Samples of *A. vulgaris, P. lanceolata* and *L. perenne* were taken from several individuals at different sunny spots.

The procedure of pollen centrifugation experiments and weight measurements has been described in detail in our previous study (Becker and Gorb 2025). Also, this previous study provided centrifugation results for the four species *A. vulgaris, P. lanceolata, P. sylvestris* and *L. perenne* and results for the pollen grain mass of *A. vulgaris*. We now used this centrifugation method to additionally characterize the adhesion properties of *C. avellana* and used the method of weight measurements to add results for the pollen grain mass of *C. avellana, P. lanceolata, P. sylvestris* and *L. perenne*.

### SEM analysis of fresh and aged pollen

**Fig. 1:**
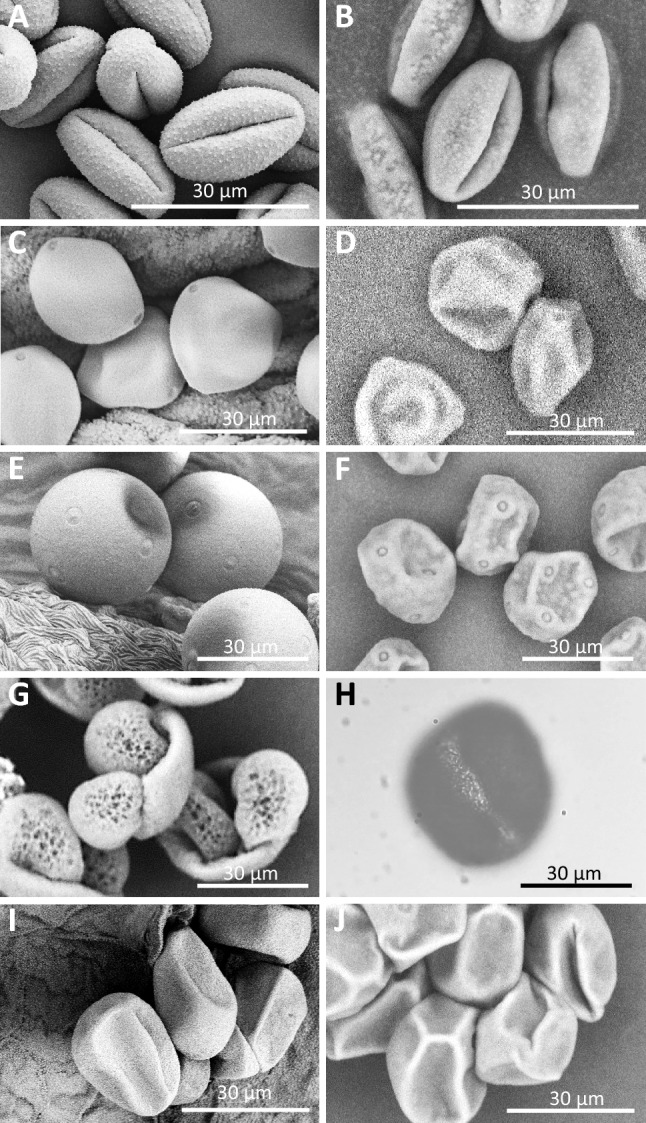
Example images of pollen grains used for volume measurements, in the order of increasing weight. **A** *A. vulgaris* fresh. **B** *A. vulgaris* aged. **C** *C. avellana* fresh. **D** *C. avellana* aged. **E** *P. lanceolata* fresh. **F** *P. lanceolata* aged. **G**,** H** *P. sylvestris* aged. **I** *L. perenne* fresh. **J** *L. perenne* aged. **A**, **C**, **E**, **I** Cryo-SEM images. **B**, **D**,** F**, **G**, **J** Room temperature SEM images. **H** Light microscopy image

Cryo-SEM analysis was done, using a Hitachi S 4800 scanning electron microscope (Hitachi High Technology, Tokyo, Japan) that was cooled with liquid nitrogen. Stigmata and stamens, as well as whole flowers from freshly cut off inflorescences were glued to a sample holder. The loaded sample holder was immediately cooled down to −140 °C in the evacuated SEM pre-chamber before being sputter coated with gold–palladium prior to examination. In addition, we used a Hitachi TM 3000 tabletop microscope (Hitachi High Technology, Tokyo, Japan) to visualize unsputtered samples from aged dry inflorescences at room temperature. Pollen grains were observed on stamen, stigma and directly on the sample holder. Examples of pollen grain images used for volume calculation are shown in Fig. 1.

### Pollen size measurements

We measured the size of random pollen grains from microscopic images, using the area analysis tool of ImageJ (version 1.54d, Wayne Rasband and contributors– National Institutes of Health, Bethesda, MD, USA). Fresh pollen grains of all species were measured from images generated during cryo-SEM analysis except for *P. sylvestris*, which was only analyzed with the Hitachi TM3000 table SEM in an aged state. Pollen grains of different age from all other species were also analyzed from images generated with the Hitachi TM3000 table SEM. In addition, aged pollen of *P. sylvestris* and fresh pollen of *A. vulgaris* were also analyzed using a Zeiss Axio Observer A1 inverse light microscope (Zeiss Group, Oberkochen, Germany). The area scale of each image in µm^2^ was calculated using a scaling slide in the case of LM images and the automatically generated scale in the case of SEM images, with respect to the current magnification.

We measured the projected particle area (PPA), determined by the outermost contour, from pollen grains in various orientations, using images of grains taken at the same magnification. Furthermore, the PPA was treated as a circular area to calculate the equivalent radius (ER) and the equivalent spherical volume of each grain. Finally, the mean value and standard deviation (SD) from each sample of pollen grains was calculated. Statistical analysis was done in Sigma Plot (version 12.5, Systat Software GmbH, Frankfurt am Main, Germany).

## Results

### Weight measurements

We analyzed ten samples from each species in both categories, fresh and aged, respectively. Fresh pollen grains were immediately weighed after sample collection. Aged pollen of *L. perenne* were aged for 6 days inside the cut-off inflorecence before measurement. Pollen of *A. vulgaris* and *P. lanceolata* were aged for 7 days, pollen of *P. sylvestris* for 8 days and pollen of *C. avellana* for 10 days before measurement. Each sample consisted of several hundreds to several thousands of pollen grains. All samples were evaluated in two separate ways.

**Fig. 2:**
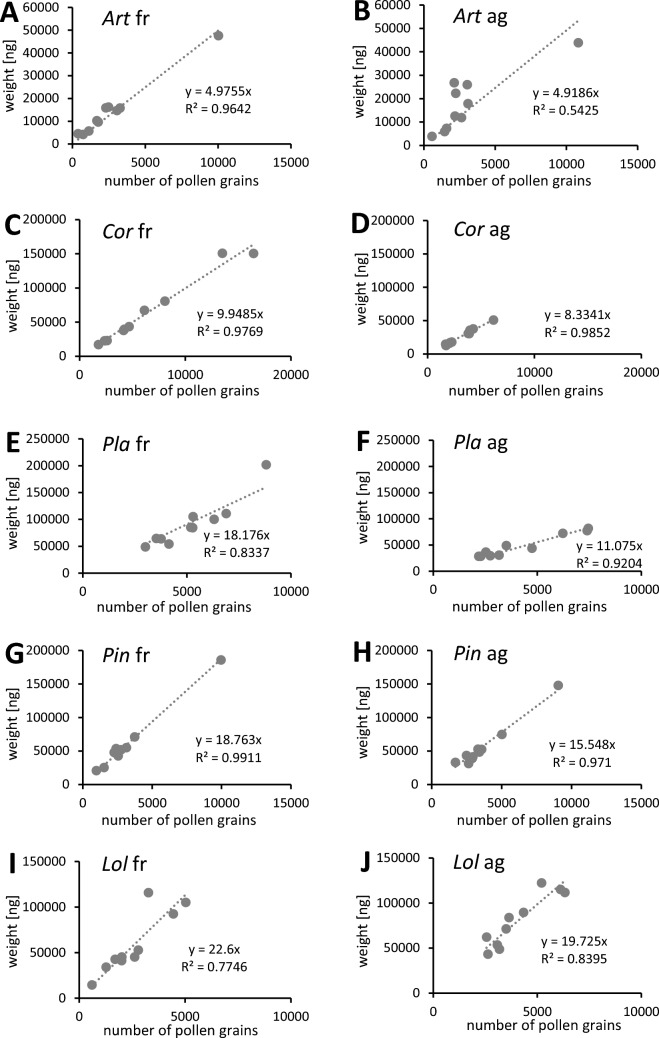
Results of weight measurements, showing the measured weight [ng] referring to the counted number of grains. **A**, **C**, **E**, **G**, **I** Fresh pollen samples, **B**, **D**, **F**, **H**,** J** Aged pollen samples. Number of samples per setting was 10. The simple linear regression (SLR) based on ordinary least square method (OLS) provides the corresponding mass of a single pollen grain and the certainty of the measurements (R^2^). fr, fresh; ag, aged; *Art*, *A. vulgaris*; *Cor*, *C. avellana*; *Pla*, *P. lanceolata*; *Pin*, *P. sylvestris*; *Lol*, *L. perenne*; abbreviations also used in further figures

For the first way of measurement, we plotted the measured mass in ng (10^–12^ kg) of every single sample against the number of grains and made a simple linear regression analysis over ten samples. The slope of the regression line was taken as the average mass of a single pollen grain and the upper and lower 95% confidence interval (CI) of the slope was calculated. This was done for each species in fresh and aged states separately. Graphs for regression analysis are shown in Fig. 2. To enable the calculation of density, the provided results for single grain mass were then expressed in pg (10^–15^ kg). With this method, in order of increasing mass, our previous study provided an average single grain mass of 4976 ± 458 pg (slope ± CI) and 4919 ± 1475 pg for fresh and aged *A. vulgaris* pollen, respectively (Becker and Gorb 2025). In addition, our current results provide a mass of 9949 ± 687 pg and 8334 ± 314 pg for *C. avellana* fresh and aged, respectively. For *P. lanceolata,* we measured a mass of 18,176 ± 2365 pg (fresh) and 11,075 ± 982 pg (aged). For *P. sylvestris*, we measured 18,763 ± 808 pg (fresh) and 15,548 ± 1000 pg (aged) and, finally, *L. perenne* had the highest single grain mass of 22,600 ± 3974 pg (fresh) and 19,725 ± 1957 pg (aged). The values are also provided in Suppl. Table S1. The coefficient of determination (*R*^2^) ranged from 0.7746 (*L. perenne* fresh) to 0.9911 (*P. sylvestris* fresh), indicating a quite valid certainty for this method in general. The only exception was *A. vulgaris* aged with an *R*^2^ of only 0.5425. Considering the graph, this was due to three samples with a remarkably higher average single grain mass than the others.

**Fig. 3:**
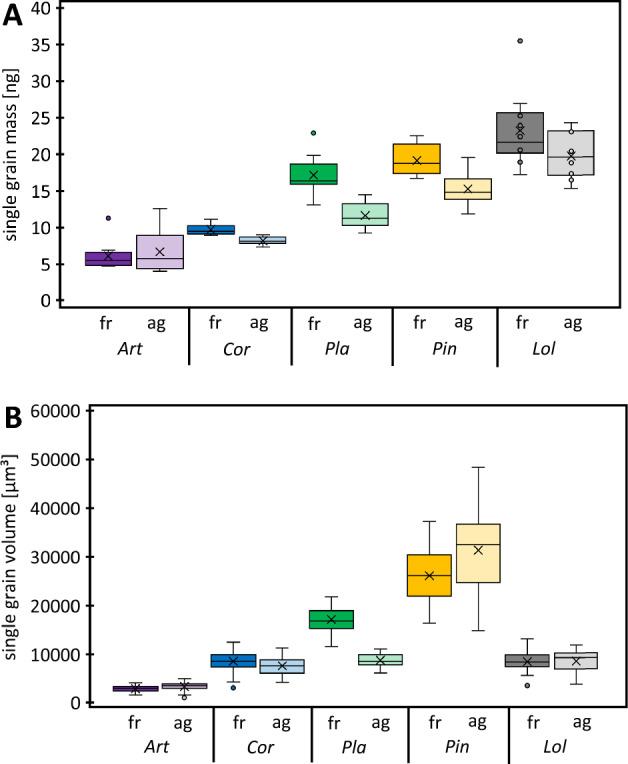
Summary of results from weight and volume measurements. **A** Results of weight measurements, showing the distribution of single grain mass [ng] for each species in fresh and aged state, based on mean values and standard deviation (SD) of each sample (*n* = 10 for every setting). **B** Results of volume calculation, showing the distribution of single grain volume [µm^3^] based on projected particle area (PPA) measurements

For the second way of evaluation, we calculated the single grain mass for each sample separately and considered mean value and SD over ten samples as the average single grain mass for statistical analysis. With this method, we measured a single grain mass of 6139 ± 1966 pg and 6696 ± 2869 pg for fresh and aged *A. vulgaris* grains, respectively. For *C. avellana,* we measured 9737 ± 779 pg (fresh) and 8210 ± 508 pg (aged). For *P. lanceolata* we measured 17,188 ± 2653 pg (fresh) and 11,684 ± 1769 pg (aged). Furthermore, we measured 19,158 ± 2034 pg (fresh) and 15,293 ± 2177 pg (aged) for *P. sylvestris* and again *L. perenne* had the highest single grain mass of 23,251 ± 5184 pg (fresh) and 19,779 ± 3123 pg (aged), respectively. Results of mean and SD analysis are shown in Fig. 3. A statistical analysis of all five species in both categories, fresh and aged, revealed a general significant difference between all samples (Kruskal–Wallis one-way ANOVA on ranks, *n*_1–10_ = 10, DF = 9; *H* = 87.219; alpha = 0.05, *P* < 0.001). Also, Shapiro–Wilk normality test passed for all samples, except for *A. vulgaris* fresh grains. Furthermore, pairwise comparison between fresh and aged samples revealed no significant difference for *A. vulgaris* (Mann–Whitney *U* rank sum test, *n*_1_, *n*_2_ = 10, *U* = 49, *t* = 106, alpha = 0.05, *P* = 0.970) and *L. perenne* (t-test, *n*_1/2_ = 10_,_ DF = 18, *t* = 1.814, alpha = 0.05, *P* = 0.086). On the other hand, *C. avellana, P. lanceolata* and *P. sylvestris* all showed a highly significant difference in single pollen grain mass between fresh and aged samples (*t*-test, *n*_1/2_ = 10_,_ DF = 18, *t* = 5.194/5.458/4.108, alpha = 0.05, *P* < 0.001 for all three species).

A comparison of both evaluations showed that the found mean values were different from the corresponding slope values. For the majority of samples, this difference ranged from 0.27% (*L. perenne* aged) to 5.75% (*P. lanceolata* fresh), indicating a high certainty of both methods in general. From all samples, *C. avellana* had the lowest standard deviation in both, fresh and aged states. This low SD, together with the high *R*^2^ value of 0.977 and 0.985 from regression analysis and only a very small difference between mean values and slope values of only 1.5 to 2.0%, designates the results for *C. avellana* as the most valid ones of all species in this study. On the other hand, the results for *A. vulgaris* showed not only the lowest *R*^2^ value of 0.542, but also a standard deviation two to three times higher than that of all other species and a discrepancy between mean and slope of 20–25%. This was probably due to the mentioned three extreme values. We can conclude that the evaluation of mean value and SD is far more sensitive to extreme values from individual samples than the regression analysis. Therefore, we only considered slope and CI for further evaluation and density calculation.

### Centrifugation experiment

We made a re-evaluation of our centrifugation experiment described elsewhere in detail (Becker and Gorb 2025), using the average single pollen grain mass from the regression analysis of our weight measurements, and included the new centrifugation results of *C. avellana* into further analysis. From the experiment consisting of over ten rounds of increasing rotation speed (1000, 2000, …, 10,000 rpm, which corresponds to an acceleration of 74–7378* g*), we counted the number of detached grains for each round and the remaining grains after round 10. Each species was analyzed in the three categories (1) fresh grains, (2) grains that were aged not in contact with the tested substrate and (3) grains aged in contact with the substrate, which in our experiment was always glass. Glass was used as a standardized substrate to enable comparison of observed adhesion forces and correlation to species-specific morphological grain properties. Using the following equation

$${{\boldsymbol{F}}}_{{\boldsymbol{c}}}=4{{\boldsymbol{\pi}}}^{2}\times {\boldsymbol{r}}\times {{\boldsymbol{n}}}^{2}\times {\boldsymbol{m}}={{\boldsymbol{F}}}_{{\boldsymbol{A}}}$$

with ***r*** as the distance between pollen grains and the center of the centrifuge = 0.066 m, ***n*** as the rotation in rev*s^−1^ and ***m*** as the single pollen grain mass in µg (10^–9^ kg), we calculated the actual centrifugal force ***F***_***c***_ in nN, which is equivalent to the adhesion force (***F***_***A***_) of a pollen grain in the moment of its detachment. Adding the weight force of a single pollen grain (***F***_***g***_ = ***m*** × ***g*** with ***g*** = 9.81 m/s^2^), we also calculated the safety factor (***S***_***F***_), which is given by

$$S_{F} = \, F_{c} /F_{g}$$

**Fig. 4:**
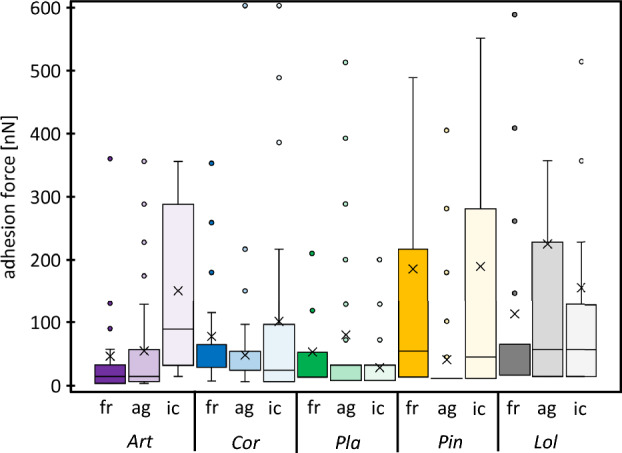
Evaluation of centrifugation experiments, comparing the distribution of adhesion force [nN] for all species in the three categories fresh, aged, and aged in contact with the substrate. Values above 600 nN (uppermost 4% of data) are not shown. A full version of this graph is provided in the supplementary materials (Fig. S2). Corresponding safety factors are shown in Fig. S1. Data is shown in Suppl. Table S1 and S2. ic, in contact; abbreviation also used in further figures

Thus, we calculated the maximal adhesion force (***MF***_***A***_) and the maximal safety factor (***MS***_***F***_) for each single detached pollen grain. For the remaining grains, the force calculated for round 10 was used. However, as the number of pollen grains strongly varied between samples, we only evaluated the percentage of detached grains for each round and remaining grains, respectively, which is equivalent to the detachment probability, resulting in a comparable sample size of 99–101 force values (due to adjustment to integers) for each sample. As Shapiro–Wilk normality test failed for all samples, we chose the median value as the primary descriptive parameter. The resulting force distributions up to 600 nN are summarized in Fig. 4 and Suppl. Table S2.

In detail, for *A. vulgaris* we measured a median *MF*_*A*_ of 14.4 nN for fresh grains and a similar *MF*_*A*_ of 14.2 nN for aged grains, while the grains that aged in contact with the substrate had a significantly higher *MF*_*A*_ of 89.0 nN (Kruskal–Wallis one-way ANOVA on ranks, *n*_1,2_ = 100, *n*_3_ = 101, DF = 2; *H* = 72.497; alpha = 0.05, *P* < 0.001, pairwise comparison done with Dunn´s method). For *C. avellana*, fresh grains had a significantly higher *MF*_*A*_ of 64.8 nN compared to both, aged grains with *MF*_*A*_ of 24.1 nN and in contact grains with an equal *MF*_*A*_ of 24.1 nN (Kruskal–Wallis one-way ANOVA on ranks, *n*_1_ = 100, *n*_2,3_ = 99, DF = 2; H = 30.650; alpha = 0.05, *P* < 0.001, Dunn´s method). In a similar way, for *P. lanceolata*, we found a significantly higher *MF*_*A*_ of 13.2 nN for fresh grains compared to aged and in contact ones with 8.0 nN, respectively (Kruskal–Wallis- One-Way-ANOVA on ranks, *n*_1_ = 99, *n*_2_ = 100, *n*_3_ = 99, DF = 2; H = 27.404; alpha = 0.05, *P* < 0.001, Dunn´s method). For *P. sylvestris*, we found *MF*_*A*_ of 54.3 nN for fresh grains. For aged grains, we found a lower *MF*_*A*_ of 11.3 nN and in contact grains had again a higher *MF*_*A*_ of 45.0 nN. All three values were significantly different from each other (Kruskal–Wallis one-way ANOVA on ranks, *n*_1_ = 99, *n*_2_ = 101, *n*_3_ = 99, DF = 2; *H* = 100.327; alpha = 0.05, *P* < 0.001, Dunn´s method). Finally, fresh *L. perenne* grains had *MF*_*A*_ of 65.4 nN, while aged and in contact grains both had *MF*_*A*_ of 57.1 nN. Interestingly, the only significant difference was between fresh and in contact grains (Kruskal–Wallis one-way ANOVA on ranks, *n*_1,2_ = 100, *n*_3_ = 99, DF = 2; *H* = 9.532; alpha = 0.05, *P* = 0.009, Dunn´s method).

Corresponding maximal safety factor (*MS*_*F*_) values are shown in Suppl. Table S3 and Fig. S1. A diagram considering the whole force distribution beyond 600 nN is shown in Fig. S2. Considering the *MS*_*F*_, the significant difference in all species, except for *A. vulgaris*, was further reduced. Only for *P. sylvestris*, there was a significant difference between fresh and aged grains as well as between aged and in contact ones (Kruskal–Wallis one-way ANOVA on ranks, *n*_1_ = 100, *n*_2_ = 101, *n*_3_ = 99, DF = 2; *H* = 64.586; alpha = 0.05, *P* < 0.001, Dunn´s method).

**Fig. 5:**
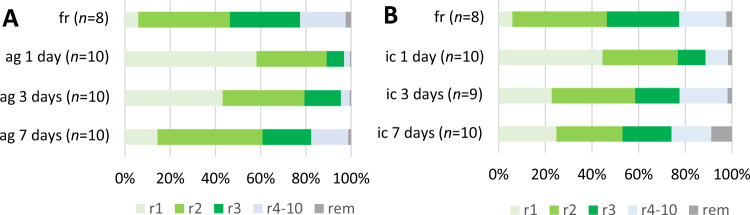
Summary of adhesion performance of pollen grains from detailed centrifugation analysis on *C. avellana*, showing the percentage of detached grains per round for every setting. **A** Summary for aged pollen. **B** Summary for pollen aged in contact with the substrate. r1–r10, round 1–round 10; rem, remaining grains after round 10

In order to further understand the effect of pollen grain age on the measured adhesion force and to compare it to our previous results, we made a more detailed centrifugation analysis of *C. avellana* in a similar way as previously described for *A. vulgaris* (Becker and Gorb 2025). Therefore, the categories “aged” and “in contact” were further subdivided into “1 day”, “3 days” and “7 days”, resulting in seven categories in total. For each category, we had eight to ten samples as simultaneous replications. The mean values of all sample replications per category were used for evaluation. We compared the percentage of detached grains in round 1, round 2 and round 3, as well as the summarized percentage of detached grains in rounds 4–10 and the remaining grains after round 10 between all categories. The results are shown in Fig. 5. Following this evaluation, there was a remarkable difference between fresh and aged grains and also between fresh and in contact grains, while the difference between aged grains and in contact grains in general was mainly present at low rotation speed (round 1) and high speed (rounds 4–10 and remaining grains). A detailed description of these results is provided as an additional paragraph in the Suppl. Data S1. Based on these results, one can conclude that the adhesion of *C. avellana* pollen grains decreased during the first day for both, the aged and the in contact grains and was lowest after 1 day. Afterward the adhesion increased again, while this increase was stronger for pollen in contact than for aged ones.

We made a statistical analysis of the differences between all categories described above. Due to simultaneous replications, we had a sample size of *n* = 10 for almost all categories. Only for fresh grains and for in contact 3 days, the sample size was reduced to *n* = 8 and *n* = 9, respectively. Shapiro–Wilk normality test failed for some samples; therefore, we used the non-parametric Kruskal–Wallis one-way ANOVA on ranks for analysis. The differences in round 1 were significant for all seven categories (ANOVA on ranks, *n*_1–7_ = 8–10; DF = 6: *H* = 13.246, alpha = 0.05, *P* = 0.039). In contrast, there were no significant differences in round 2 (ANOVA on ranks, *n*_1–7_ = 8–10; DF = 6: *H* = 3.489, alpha = 0.05, *P* = 0.745) as well as in round 3 (ANOVA on ranks, *n*_1–7_ = 8–10; DF = 6: *H* = 8.697, alpha = 0.05, *P* = 0.191). The differences in rounds 4–10 were again highly significant (ANOVA on ranks, *n*_1–7_ = 8–10; DF = 6: *H* = 30.204, alpha = 0.05, *P* < 0.001), as well as the differences among the remaining grains (ANOVA on ranks, *n*_1–7_ = 8–10; DF = 6: *H* = 29.712, alpha = 0.05, *P* < 0.001). This underlines that the categories fresh, aged and in contact in *C. avellana* differed from each other mainly in the range of very low and very high adhesion, while moderate adhesion was similar for all categories.

### Volume measurements

We used the projected particle area (PPA) measured from 2D SEM images to calculate the single grain volume for each species in fresh and aged state. Depending on the number of suitable images, the number of analyzed grains differed between species and category. In total, the analysis with this method included 36 fresh and 96 aged *A. vulgaris* pollen, which was the largest sample size of all species studied. For *C. avellana*, we analyzed 61 fresh and 50 aged grains. For *P. lanceolata*, we analyzed 54 fresh grains that were in fact completely hydrated and 50 aged grains. For *P. sylvestris*, only 50 aged grains were analyzed with SEM and finally, *L. perenne* had the lowest sample size with only 17 fresh and 14 aged grains. The results are shown in Fig. 3B.

Based on this analysis, we calculated an average single grain volume of 2764.925 ± 647 µm^3^ and a corresponding equivalent radius (ER) of 8.707 ± 0.713 µm for fresh *A. vulgaris* grains. For aged *A. vulgaris* grains, the analyzed sample resulted in an average volume of 3240.951 ± 730.834 µm^3^. However, a statistical analysis showed that this sample, in contrast to all others, was not normal distributed, but strongly right shifted instead, indicating an overestimation of the average volume. Therefore, this result cannot be regarded as representative. Considering only the minimal and maximal values (961.085 µm^3^ and 4832.544 µm^3^), the mean value of both indicates a probable true average volume of 2896.841 µm^3^, which lies within the range of the provided fresh grain volume. We also analyzed 20 hydrated grains, resulting in a remarkably similar volume of 2991.595 ± 381.017 µm^3^ and an ER of 8.939 ± 0.389 µm.

For *C. avellana*, we found a fresh grain volume of 8618.347 ± 1855.329 µm^3^ and a corresponding ER of 12.719 ± 0.985 µm. Accordingly, we found a slightly lower volume of 7668.015 ± 1712.086 µm^3^ and an ER of 12.233 ± 0.931 µm for aged grains. For *P. lanceolata*, the analyzed hydrated grains provided a volume of 17,158.540 ± 2416.654 µm^3^ and an ER of 16.000 ± 0.759 µm. The volume of aged grains was much lower, with a mean value of 8808.668 ± 1242.223 µm^3^ and an ER of 12.812 ± 0.613 µm, resulting in *P. lanceolata* showing the largest discrepancy of single grain volume between fresh and aged grains among the examined species. The analysis of *P. sylvestris* aged grains provided the largest volume of all examined grains with 26,142.849 ± 5317.015 µm^3^ and an ER of 18.411 ± 1.253 µm. Finally, for *L. perenne* fresh grains, we found a volume of 8538.255 ± 2170.794 µm^3^ and an ER of 12.580 ± 1.175 µm, which was even slightly smaller than the aged grain volume of 8653.326 ± 2247.326 µm^3^ with an ER of 12.736 ± 1.239 µm. (Fig. 3B) This indicates that despite being a freshly collected sample analyzed with cryo-SEM, the measured fresh pollen grains were already in a similar state as the aged grains and therefore probably not representative for the true fresh state of *L. perenne* in general. The results are also summarized in Suppl. Table S1.

The analysis of 65 aged *P. sylvestris* grains from LM images resulted in an average single grain volume of 31,385.085 ± 7615.539 µm^3^, which was 33.6% larger than the provided grain volume from SEM images, despite accurately considering the scale bar. The underlying PPA was 12.7% larger and the corresponding ER of 19.568 ± 1.640 µm was 6.8% larger than for grains from SEM images. To further examine this discrepancy, we analyzed 18 fresh *A. vulgaris* grains from another LM image, resulting in a discrepancy of 44.8% in volume, 28.7% in PPA and 10.6% in ER compared to the provided results from cryo-SEM images. We conclude that the conditions of both methods lead to a discrepancy in the measured PPA. As a consequence, a comparison of LM images to SEM images must be treated with caution for this analysis method and was therefore not considered in our evaluation. Furthermore, we made a second analysis of pollen grains from SEM images of different magnifications, resulting in similar results for all species in the fresh state.

Statistical analysis of our results provided further important insights. While the *A. vulgaris* aged sample was the only one which was not normally distributed, the equal variance test failed in some further cases. Therefore, we used non-parametric tests for analysis of those samples. This analysis revealed a significant difference between fresh and aged *A. vulgaris* grains (Mann–Whitney *U* rank sum test, *n*_1_ = 36, *n*_2_ = 96, *U* = 1001, *t* = 1667, alpha = 0.05, *P* < 0.001), which means that the measured aged grain volume was significantly higher than the fresh grain volume, while the shape of the grains, according to our observation, did not change at all. This fact further underlines an overestimation of the aged grain volume. The additional analysis of hydrated grains also showed a significant difference to aged grains (Mann–Whitney U rank sum test, *n*_1_ = 96, *n*_2_ = 20, *U* = 614, *t* = 824, alpha = 0.05, *P* = 0.012), but surprisingly not to fresh ones (Mann–Whitney *U* rank sum test, *n*_1_ = 36, *n*_2_ = 20, *U* = 291, *t* = 639, alpha = 0.05, *P* = 0.241), despite obvious changes in shape.

For *P. sylvestris*, we found a similar difference as for *A. vulgaris*, with the measured aged grain volume from LM images being significantly higher than the aged grain volume from SEM images (Mann–Whitney U rank sum test, *n*_1_ = 50, *n*_2_ = 65, *U* = 947, *t* = 2222, alpha = 0.05, *P* < 0.001). This further underlines the conclusion that LM images lead to an overestimation of grain volume. In contrast, for *C. avellana* the aged grain volume was significantly lower than the fresh grain volume (t-test, *n*_1_ = 61_,_ *n*_2_ = 50, DF = 109, *t* = 2.779, alpha = 0.05, *P* = 0.006), proving these results to be valid. A similar result was found for *P. lanceolata* (Mann–Whitney *U* rank sum test, *n*_1_ = 54, *n*_2_ = 49, *U* = 0.000, *t* = 1225, alpha = 0.05, *P* < 0.001). On the other hand, the analysis of *L. perenne* grains provided no significant difference between the measured fresh and aged grains (*t*-test, *n*_1_ = 17_,_ *n*_2_ = 14, DF = 29, *t* = −0.145, alpha = 0.05, *P* = 0.886), further underlining the assumption that their state of age was in fact similar. In addition, we statistically compared the measured volume from images with a different magnification. As a result, only one out of six comparisons showed a significant difference. This was the comparison of hydrated *A. vulgaris* grains with 450 × and 800 × magnifications, respectively (*t*-test, *n*_1_ = 26_,_ *n*_2_ = 20, DF = 44, *t* = −3.878, alpha = 0.05, *P* < 0.001). However, this difference was probably caused rather by variation within the actual samples than by the used magnification.

### Density calculation

For pollen grain density calculation, we combined the results of our weight measurements in pg, using slope and CI from regression analysis and our volume measurements in µm^3^, using mean and SD, for both, fresh and aged pollen from analysis of SEM images. From our volume measurements, the samples of *A. vulgaris* aged and *L. perenne* fresh were not representative, as previously described, and could not be considered for density calculations. Furthermore, we were confronted with the problem that the data sets for mass and volume were collected in two separate experiments. Thus, the water content of weighed fresh pollen might be different from that of fresh pollen analyzed with the cryo-SEM method that were used for volume measurements. While the *P. lanceolata* fresh grains used for volume measurements were completely hydrated, we assume a lower water content for the weighed fresh pollen due to the experimental conditions. As a consequence, the measured hydrated grains were added as a separate third category for *P. lanceolata*.

Based on these decisions, the volume samples directly used for density calculation were *A. vulgaris* fresh, *C. avellana* fresh, *C. avellana* aged, *P. lanceolata* hydrated, *P. lanceolata* aged, *P. sylvestris* aged (SEM) and *L. perenne* aged. Furthermore, we made the assumption that the measured differences in pollen grain mass were caused only by the evaporation of water and were therefore equivalent to the difference in volume. Thus, representative values for the pollen grain volume of the missing categories can be calculated, using the measured values of the remaining volume categories and the measured differences in pollen grain mass. In the same way, the mass of *P. lanceolata* hydrated grains was calculated. However, the mean values added with this method come without SD and CI. Also, one need to consider that we assumed no additional air volume to be present in the grains due to the observed changes in shape.

Considering all these preconditions, we provide the following results, also summarized in Suppl. Table S1 and Fig. S3. For *A. vulgaris* fresh grains, we found an average mass of 4975 ± 458 pg and an average volume of 2765 ± 647 µm^3^, resulting in a density of 1.800 pg/µm^3^, which is equivalent to 1.800 g/cm^3^ (1800 kg/m^3^), and for aged grains, we found a mass of 4918 ± 1475 pg and an assumed volume of 2708 µm^3^, resulting in a density of 1.816 g/cm^3^. For *C. avellana* fresh grains, we found a mass of 9948 ± 687 pg and a volume of 8618 ± 1855 µm^3^, resulting in a lower density of 1.154 g/cm^3^, compared to *A. vulgaris*, and for aged grains, we found a mass of 8334 ± 313 pg and a volume of 7668 ± 1712 µm^3^, resulting in a density of 1.087 g/cm^3^. For *P. lanceolata*, we provide an assumed mass for hydrated grains of 19,425 pg and a measured volume of 17,159 ± 2417 µm^3^, resulting in a density of 1.132 g/cm^3^, while fresh grains had a mass of 18,176 ± 2365 pg and a corresponding assumed volume of 15,910 µm^3^, resulting in a slightly higher density of 1.142 g/cm^3^. Aged grains had a mass of 11,075 ± 928 pg and a volume of 8809 ± 1242 µm^3^, resulting in a density of 1.257 g/cm^3^. Furthermore, we measured a mass of 18,763 ± 808 pg and calculated a volume of 29,358 µm^3^ for fresh *P. sylvestris* grains, resulting in a rather low density of 0.639 g/cm^3^, while aged grains had a mass of 15,548 ± 1000 pg and a volume of 26,142 ± 5317 µm^3^, resulting in the lowest density of all with 0.595 g/cm^3^. Finally, the analysis of *L. perenne* provided a fresh grain mass of 22,600 ± 3974 pg and a corresponding assumed volume of 11,528 µm^3^, resulting in a high density of 1.960 g/cm^3^ and an aged grain mass of 19,725 ± 1957 pg, together with a volume of 8653 ± 2248 µm^3^, resulting in the highest density of all examined samples with a value of 2.279 g/cm^3^.

### Data collection from the literature

In addition to our own results, we made a comprehensive literature research to collect references providing further data on pollen grain mass, size (provided as diameter, radius or volume) and density. We only considered references that provided results of at least two of these parameters, so the missing one could be easily calculated. In one study (Vaissière and Vinson 1994), only the mass and number of grains of a larger sample were provided. In this case, the single grain mass of the species in question (*Gossypium hirsutum*) was estimated from these values. Volume calculation was done in the following way: if only one diameter (***d***) was given, we used the corresponding radius (*r* = *d*/2) to calculate the equivalent spherical volume (***V***) as

$$V = \left( {4/3} \right)*\pi *r^{3}$$

If two diameters were provided, we used the adapted formula

$$V = \left( {4/3} \right)*\pi *a^{2} *b,$$

with ***a*** being the smaller radius and ***b*** being the larger one (Stead et al. 1979). In the same way, the equivalent spherical radius and diameter were calculated from a given volume. Finally, results for single grain mass were expressed in pg and results for single grain volume were expressed in µm^3^, while density values were expressed in g/cm^3^. An overview over all considered references and the methods used there is given in Suppl. Table S4. The resulting data collection is provided in Suppl. Table S5, with data directly provided by the references marked in gray. (Durham 1946; Crawford 1949; Gilissen 1977; Luu et al. 1997; Aylor 2002; Sofiev et al. 2006; Urzay et al. 2009; Gómez-Noguez et al. 2022). However, one need to consider that this data collection is biased and conclusions taken from it need to be treated with caution. In addition to the 11 results of our own, the literature research provided 84 further data points, resulting in 95 mass and volume values in total for more than 70 species (some species have been analyzed more than once). We collected 47 values for eudicots, 13 values for monocots and 35 values for gymnosperms and plotted these data samples in mass/volume graphs to calculate the average density, using simple linear regression. However, one value belonging to the species *Gossypium hirsutum* (Malvaceae, eudicots) and three values belonging to *Zea mays* (Poaceae, monocots) had to be treated separately, as these grains were ten times larger and heavier than all other species in their corresponding groups. Therefore, these four values were considered for statistical analysis, but excluded from the graphs. Also, all referred monocot species belonged to the Poaceae family except for one value of *Butomus umbellatus* (Butomaceae), making the data sample representative for Poaceae, but probably not for monocots in general. Furthermore, the gymnosperm data sample was also biased as all species belonged to the order Coniferales, with 12 out of 35 species belonging to the genus *Pinus* (Pinaceae) and 10 other species to the genus *Abies* (Pinaceae), which has also very large grains compared to the others. We separated the data sample into small (*n* = 21) and large (*n* = 14) gymnosperm pollen below and above a volume of 160,000 µm^3^ for further analysis.

**Fig. 6:**
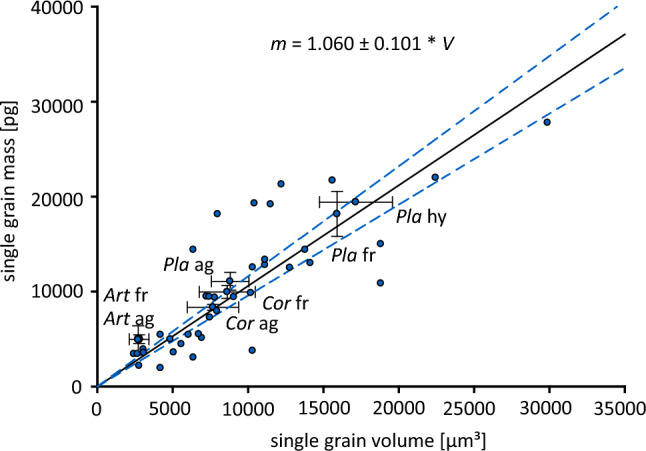
Results of simple linear regression analysis (SLR) for pollen of eudicots without *G. hirsutum*, based on own measurements and literature data, showing the correlation between single grain volume [µm.^3^] (x-axis) and single grain mass [pg] (y-axis). It provides the average grain density as slope (black line) ± 95% confidence interval (CI) (blue dashed lines). Error bars show variation found in our own results. Vertical error bars show 95% CI of weight measurements. Horizontal error bars show SD of volume calculation. Corresponding data is provided in Suppl. Table S3 and S5

**Fig. 7:**
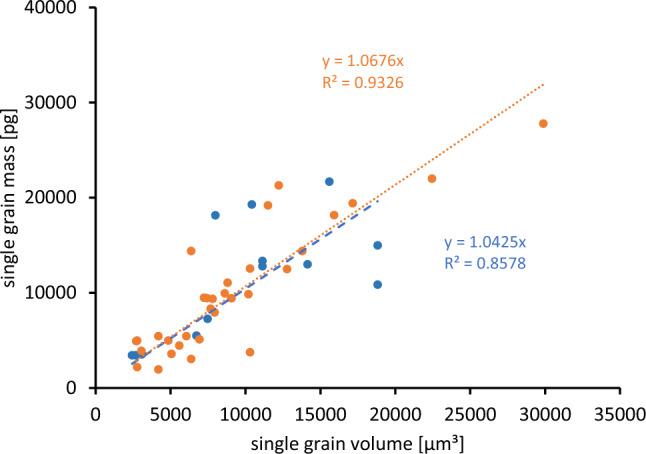
Results of simple linear regression analysis (SLR) for pollen of insect-pollinated (*n* = 14) and wind-pollinated (*n* = 32) eudicots without *G. hirsutum*, based on literature data, showing the correlation between single grain volume [µm^3^] (x-axis) and single grain mass [pg] (y-axis). It provides the average grain density as slope. Blue data dots indicating insect-pollinated species and orange data dots indicating wind-pollinated species

In addition to simple linear regression analysis, we also calculated the 95% CI for each plot. As a result, the collected data for eudicots provided an average pollen grain density of 1.06 ± 0.10 g/cm^3^ (slope ± CI) (Fig. 6). We distinguished between insect- and wind-pollinated eudicots. For the 14 values of insect-pollinated species (*G. hirsutum* excluded), regression analysis provided an average grain density of 1.04 g/cm^3^ and for the 32 values of wind-pollinated species, including our results, we found a value of 1.07 g/cm^3^ (Fig. 7). Statistical comparison between both samples showed no significant difference in single grain mass, volume or density, whether *G. hirsutum* was included (*n* = 15) or not (*n* = 14) (ANOVA on ranks, *n*_1_ = 15; *n*_2_ = 14; *n*_3_ = 32; DF = 2: H = 0.610/0.609/2.943, alpha = 0.05, *P* = 0.737/0.737/0.230).

**Fig. 8:**
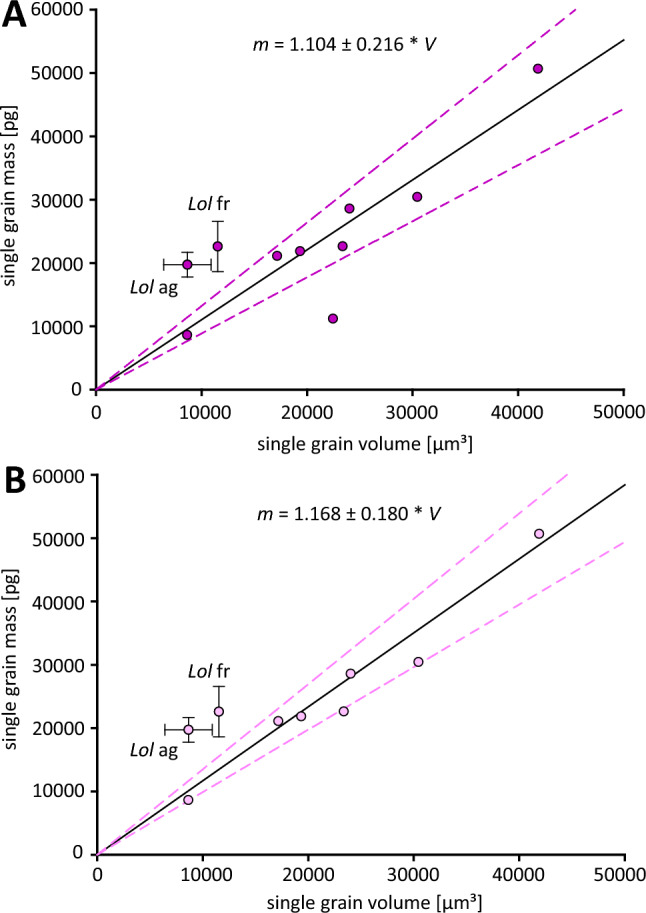
Results of simple linear regression analysis (SLR) for pollen of monocots without *Zea mays*, based on own measurements and literature data, showing the correlation between single grain volume [µm.^3^] (x-axis) and single grain mass [pg] (y-axis). It provides the average grain density as slope (black line) ± 95% confidence interval (CI) (colored dashed lines) Error bars show variation found in our own results. Vertical error bars show 95% CI of weight measurements and horizontal error bars show SD of volume calculation. **A** All data for monocots. **B** Only Poaceae data without *Butomus umbellatus*. Corresponding data is provided in Suppl. Table S3 and S5

**Fig. 9:**
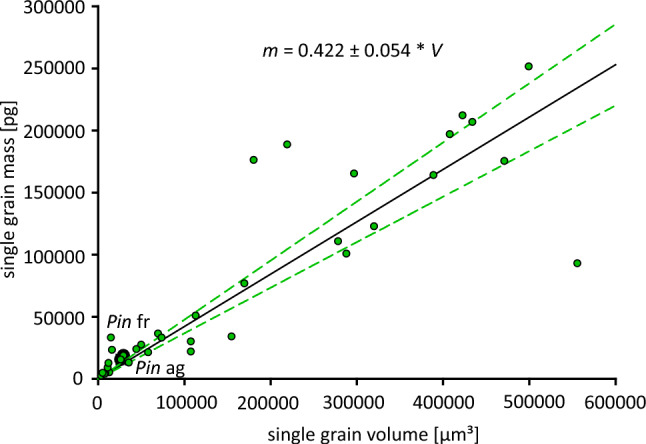
Results of simple linear regression analysis (SLR) for pollen of gymnosperms, based on own measurements and literature data, showing the correlation between single grain volume [µm.^3^] (x-axis) and single grain mass [pg] (y-axis). It provides the average grain density as slope (black line) ± 95% confidence interval (CI) (green dashed lines). Bold-framed dots mark the position of our results for *P. sylvestris*. Error bars are not visible at this scale. Corresponding data is provided in Suppl. Table S3 and S5

**Fig. 10:**
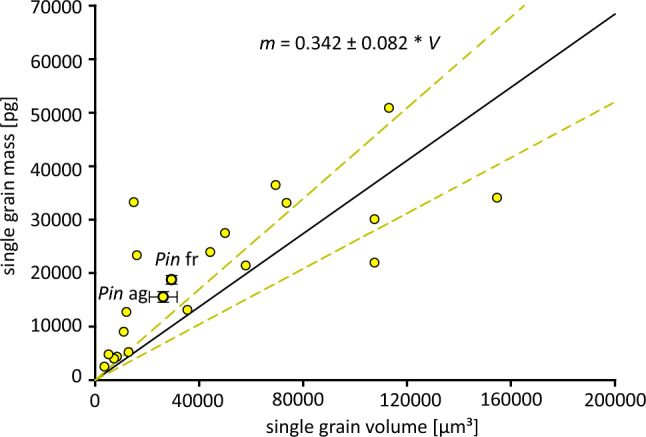
Results of simple linear regression analysis (SLR) for small pollen of gymnosperms below a volume of 160,000 µm^3^, based on own measurements and literature data, showing the correlation between single grain volume [µm.^3^] (x-axis) and single grain mass [pg] (y-axis). It provides the average grain density as slope (black line) ± 95% confidence interval (CI) (yellow dashed lines). Error bars show variation found in our own results. Vertical error bars show 95% CI of weight measurements and horizontal error bars show SD of volume calculation. Corresponding data is provided in Suppl. Table S3 and S5

**Fig. 11:**
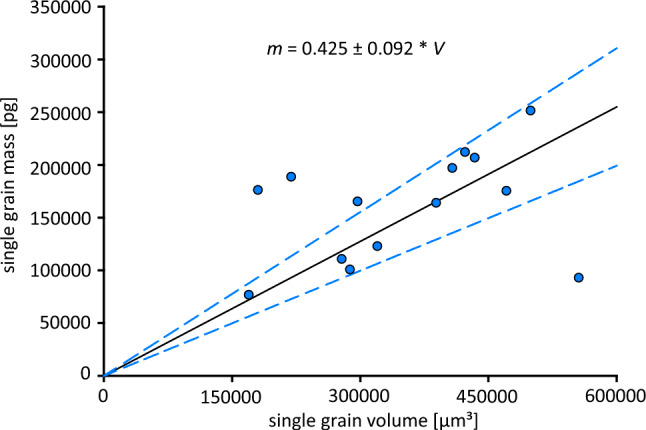
Results of simple linear regression analysis (SLR) for large pollen of gymnosperms with a volume above 160,000 µm^3^, based on literature data, showing the correlation between single grain volume [µm.^3^] (x-axis) and single grain mass [pg] (y-axis). It provides the average grain density as slope (black line) ± 95% confidence interval (CI) (blue dashed lines). Corresponding data is provided in Suppl. Table S5

**Fig. 12:**
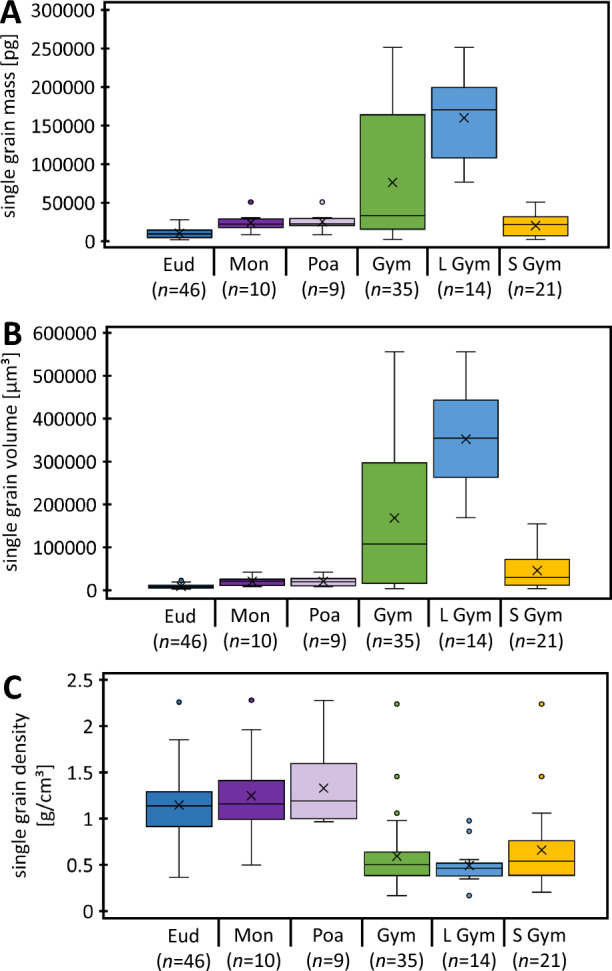
Summary of all own results and literature data, comparing the distribution of single grain mass (**A**), volume (**B**) and density (**C**) between all provided samples, with *G. hirsutum* and *Z. mays* excluded (see Fig. 6–11). Corresponding data is provided in Suppl. Table S3 and S5. Eud, eudicots sample; Mon, monocots sample; Poa, Poaceae sample; Gym, gymnosperm sample; containing all pollen; L Gym, large gymnosperm pollen sample; S Gym, small gymnosperm pollen sample; abbreviations also used in further supplementary figures

For the ten values belonging to monocots (*Z. mays* excluded), regression analysis provided an average pollen grain density of 1.104 ± 0.216 g/cm^3^ (slope ± CI) (Fig. 8A), which was quite comparable to that of eudicots. Additionally, excluding *B. umbellatus* and considering only the remaining nine values of Poaceae species resulted in a slightly higher average density of 1.168 ± 0.180 g/cm^3^ (Fig. 8B). For all 35 values of gymnosperms, we found an average grain density of 0.422 ± 0.054 g/cm^3^ (Fig. 9). However, splitting the gymnosperm sample into small (< 160,000 µm^3^) and large pollen (> 160,000 µm^3^) showed that large pollen had an average density of 0.425 ± 0.092 g/cm^3^ (Fig. 10), while for small pollen, we found a lower average density of 0.342 ± 0.082 g/cm^3^ (Fig. 11). In general, for all results, the 95% confidence interval provided an uncertainty of ± 10–25% of the calculated average pollen grain density. As it can be seen, all results of pollen density were remarkably lower for gymnosperms than for angiosperms. To further examine this, we made a statistical comparison of all samples, treating monocots and Poaceae as separate samples, as well as the three gymnosperm samples, resulting in six samples in total (Fig. 12A, B, C). According to this analysis, the density of gymnosperm pollen was indeed significantly lower than the density of angiosperm pollen, while on the other hand, there was no significant difference between eudicots and monocots or Poaceae, and also between small or large gymnosperms (ANOVA on ranks, *n*_1–6_ = 12–47; DF = 5: H = 61.868, alpha = 0.05, *P* < 0.001; Dunn´s method). While the exclusion of *G. hirsutum* and *Z. mays* from the data samples obviously affected the distribution of mass and volume (see Suppl. Fig. S4), it had no effect on these statistical results for pollen density.

Considering the grain volume, there was first the predestinated significant difference between small and large gymnosperms, as these two samples do not overlap, but also the pollen grain volume of eudicots was significantly lower compared to all other samples, while there was no significant difference between all others. The same result was found for the pollen grain mass (ANOVA on ranks, *n*_1–6_ = 12–47; DF = 5: *H* = 69.370/61.270, alpha = 0.05, *P* < 0.001; Dunn´s method). Also, after excluding *G. hirsutum* and *Z. mays*, as well as the large gymnosperms sample, comparing the remaining angiosperms to the small gymnosperms still provided the same result that pollen of eudicots were significantly smaller and lighter than all others, while there was no significant difference between monocots and small gymnosperms (ANOVA on ranks, *n*_1–4_ = 9–46; DF = 3: *H* = 29.909/26.143, alpha = 0.05, *P* < 0.001; Dunn´s method). Further graphs, considering only the literature data are shown in Suppl. Fig. S5-S7.

## Discussion

In this study, we used the weigh-and-count approach to measure the mass of single pollen grains and the PPA, obtained from SEM images, as a basis for single grain volume calculation. Both methods were established in several studies during the last decades (Van Hout and Katz 2004; Bunderson and Levetin 2015; Galveias et al. 2022). However, to the best of our knowledge, the methods themselves have rarely been discussed yet. Our original results for eudicots and gymnosperms fit rather well to the summarized values collected from the literature (see Suppl. Table S3 and S5). Only our results for the grass species *L. perenne* differ from earlier reports for other grass species. This difference is caused by our volume measurements, while the measured mass fits well. In general, the analysis of pollen grain intrinsic parameters has always been a critical topic. After reviewing studies from the last century, Jackson and Lyford (1999) stated that theoretical standards for pollen grain analysis are not perfect due to measurement errors in size, mass, volume and density, as well as necessary approximations in calculations. This will be discussed in the following paragraphs.

### Changes in pollen grain mass analyzed with the weight-and-count method

The methods of our choice also came along with some difficulties. The main problem of our weight measurements was accurate counting. We always counted all grains in a weight sample directly, but pollen clumps that could not be dissolved forced us to estimate the number of grains in a clump, which probably caused the described extreme values in *A. vulgaris*. In general, there is a higher risk of underestimating the number of grains in a sample, resulting in an overestimation of single grain mass, while on the other hand, an underestimation of mass due to the overcounting is rather unlikely. However, the high accuracy of our results shows that an analysis based on several samples can counteract these potential errors. We also demonstrated that different ways of data evaluation can cause slightly different results, as we found a discrepancy between calculating the single grain mass of each sample separately and using the mean value, or making a regression analysis over all samples at once. Thus, in the task of standardizing the methods of pollen grain analysis, also the way of evaluation needs to be considered. The simple linear regression analysis proved to be a good choice for the weigh-and-count method, as it is less sensitive to overestimations in single weight samples. The 95% CI from our regression analysis varied from ± 3% to ± 17% of the slope value, with *A. vulgaris* aged being the only exception with a CI of ± 30% of slope value, probably due to the mentioned extreme values. Interestingly, this range fits rather well to the previous results. One study used a cantilever-based resonator to measure the mass of single pollen grains directly and found a standard deviation of ± 20–30% of mean value (Chan et al. 2014). Thus, this can be used as an estimation for the general uncertainty or variation of average pollen grain mass under certain stable conditions.

After all, these results provide only a snapshot, as the mass and volume of pollen grains can fluently change due to environmental conditions, mainly through dehydration or rehydration depending on relative ambient humidity (RH). This so-called harmomegathy has been extensively analyzed since the work of Wodehouse in 1935 (Bunderson and Levetin 2015). In our study, pollen grains were stored for aging at RH of 50% that was kept as constant as possible. Therefore, the observed reduced weight of aged grains, compared to fresh ones can be explained by loss of water. It is also known that the water content of fresh grains and therefore the potential loss of water differ between species (Pacini 2000; Nepi et al. 2001). The results of our weight measurements showed no significant difference between fresh and aged pollen mass for *A. vulgaris* and *L. perenne*, indicating a low water content already at the time of release from the stamen. For *L. perenne*, this result was rather unexpected, as grass pollen are described to have a high rate of water loss (Pacini 2000) and therefore only short viability (Fei and Nelson 2003). On the other hand, pollen of Pinaceae are described to be released with a low water content of less than 10% (Owens et al. 1998). Thus, the significant weight loss that we found for *P. sylvestris* was also unexpected. This indicates that the variation found through individual experiments might be higher than the overall literature data predicts. In a high humidity, pollen can also take up water and increase in weight over time. For example, Bunderson and Levetin (2015) showed that this was the case for pollen of three *Juniperus* species, while another study investigated the same phenomenon in some eudicots and monocots (Griffiths et al. 2012). In general, while we aim to provide a certain average value for estimation of pollen grain mass, the potential effect of humidity still needs to be always carefully considered.

### Effect of pollen grain mass during centrifugation experiments

The results from our weight measurements are also important for further evaluation of our centrifugation experiments. The main results of these experiments have been extensively discussed in our previous study (Becker and Gorb 2025), including all circumstances and conditions of the centrifugation method. However, the new evaluation, including the effect of pollen grain mass and calculation of the actual forces, provides some further insights. The observed potential adhesion force of single pollen grains from the examined species spread over a wide spectrum, reaching from 3 nN (*A. vulgaris* aged) up to more than 1600 nN (*L. perenne* fresh grains). However, the probability of such a high adhesion was found to be rather low, as the average adhesion over all species and categories was around 100 nN and only 57 values (4%) out of 1495 values in total were above 600 nN. Thus, in any case, adhesion conditions of individual grains can strongly differ from the majority, underlining the importance of an extensive quantitative comparison to provide certain conclusions on a species. In our previous study, we used the safety factor to exclude the effects of pollen grain mass. We found that this way of evaluation provides a good approach to compare the adhesion properties of several species under similar conditions. On the other hand, if the question is how grains of one species are affected by the experimental conditions, changes in grain mass due to dehydration also need to be considered. Comparing the median adhesion between the three categories fresh, aged, and in contact for each species, we found ten significant differences in total. However, six of these ten significant differences were caused by changes in pollen grain mass and disappeared when we considered the safety factor instead. This means, while two grains can have the same adhesion safety factor due to their surface geometry and presence of coating layers, the force necessary to detach them can still vary due to their current water content that affects mass and volume.

Another interesting aspect was the result of our centrifugation analysis on *C. avellana*. A similar detailed experiment on *A. vulgaris* in our previous study (Becker and Gorb 2025) showed a “cementing” effect, which means that the adhesion of grains in contact with the substrate increased over time, while no such an effect was found for aged grains. A similar phenomenon has also been found for the insect-pollinated species hairy cat’s ear (*Hypochaeris radicata*, Linné) (Asteraceae) and was explained by changes of pollenkitt due to aging (Huth et al. 2022). Interestingly, *C. avellana* showed no such tendency at all, meaning that the adhesion properties of grains remained rather similar, no matter whether they were aged in contact with the substrate or not. Furthermore, the amount of detached grains in the rounds 2 and 3 did not differ significantly between all categories, while both lower and higher rotation resulted in significant differences. As mentioned before, we found a strong variation of adhesion force within all samples. Thus, it is possible that this variation in a sample counteracts variation over time, leading to the result that the amount of grains detached by a moderate force stays rather constant. While we cannot tell whether this effect is also present in the stamen, it could be beneficial for the plant to provide stable pollen release over time or during changing surrounding conditions. Pollen grains of *C. avellana* are generally known to be very unsticky and to be shed in single grain units (Knoll 1936). This was also our own observation during the experiment. Based on these results, the presence of pollenkitt in this species has no effect on the adhesion performance. Nevertheless, to the best of our knowledge, the actual amount of pollenkitt and its physical and chemical properties in *C. avellana* have not been analyzed yet.

### Projected particle area and its significance for pollen grain volume calculation

The calculation of single grain volume based on PPA provides a good approach to analyze irregular shaped grains, as measuring the PPA of pollen in various orientations can compensate individual over- or underestimations, resulting in a valid average volume. However, an important precondition is that the distribution of pollen grain orientation is randomized. In the case of the ellipsoidal pollen of *A. vulgaris*, the probability of a horizontal orientation is much higher than a vertical orientation (Fig. 1). Therefore, measurements will result in an overestimation of PPA in a larger sample. This was probably the case for our *A. vulgaris* aged sample. On the other hand, actively targeting pollen with a specific orientation might also result in observer bias. Thus, only a statistically normally distributed sample provides a valid result for the average grain volume.

Another problem is the analysis of collapsed and crumpled grains. Aged grains of *C. avellana*, *L. perenne* and *P. lanceolata* had a highly concave surface (Fig. 1), which means that the measured PPA was always larger than the true cross-sectional area, independent of the orientation. Therefore, the resulting volume was probably also overestimated. Nevertheless, our results fit well to the literature data, except for the volume of *L. perenne*. In reality, however, this case was rather underestimated compared to the literature, which means that despite the problem of concave pollen, the PPA still provided a good approximation in our study. The remarkably small volume of *L. perenne* grains was probably caused by the fact that the used plant sample was not representative, as described before. Further analysis on this species might show whether our results need to be corrected. Another surprising result was the similar calculated volume of fresh and hydrated *A. vulgaris* grains, meaning that despite obvious changes in shape (Fig. S8), the average PPA was similar. So far, we cannot tell if this was caused by the selected observation method, or if the volume was indeed similar, indicating only minor water uptake. Other methods, enabling precise analysis of the volume or volume change, would be necessary to address this question.

In general, the change of pollen grain volume due to harmomegathy has also been analyzed extensively in previous studies. For example, Payne (1981) analyzed species of more than 150 genera of angiosperms and found volume reductions ranging from 4.2% to 77.6%. Also, those results did not show any correlation between volume reduction and pollen grain size or phylogeny, indicating that this is a general phenomenon in angiosperms. Furthermore, if pollen grains are treated with specific chemicals for experimental analysis, this also needs to be considered, as at least some chemicals can cause changes in pollen grain size of up to 10% (Reitsma 1969).

The comparison of SEM images to LM images showed a significantly higher PPA obtained from LM images for *P. sylvestris* and also for *A. vulgaris*. In general, in previous studies, LM was the primary method (Payne 1972; Nepi et al. 2001; Sosnoskie et al. 2009), while SEM was used less often (Niklas 1988; Pope 2010) to depict pollen for further geometrical measurements. One study, which compared both methods, stated that pollen shrink in the SEM due to vacuum conditions (Van Hout and Katz 2004), which might also have caused the observed difference for *P. sylvestris* in our study. However, this was not the case for the discrepancy in *A. vulgaris*, which was analyzed with cryo-SEM. Thus, some additional effect must have caused these differences. As the scale in SEM images is automatically calculated by the software, a calibration error can potentially cause an over- or underestimation of the size of the observed objects. However, while one cannot exclude this possibility, it is rather unlikely. Instead, we assume an overestimation of PPA in LM images due to the lower image quality (Fig. 1H).

Putting all this together, the calculation of single pollen grain volume comes with a basic uncertainty, especially if only one diameter or radius is given. In general, for calculation of a spherical volume, a variation of 10% in diameter or radius already causes 25% variation in volume. Also for our results, SD provides an overall variation of 4–10% in equivalent radius (ER) and 3–30% in volume. However, this fits with the results of previous studies. Van Hout and Katz (2004) provided results for pollen grain diameter with an SD of 4–7%. Sabban and van Hout (2011) found an SD of 2–8%, Chan et al. (2014) an SD of 4–10% and Bunderson and Levetin (2015) an SD of 10–14% for pollen grain diameter. Thus, these results provide a good benchmark for the uncertainty of pollen grain volume measurements.

### Characterization of pollen grain density

Following the described conclusions about the variation of measured pollen grain mass and volume, the calculation of density also comes along with an uncertainty of up to 30% in both dimensions (Fig. S3). Again, these results fit well to previous findings. Harrington and Metzger (1963) found standard deviations of pollen density of 10–20% of the mean value. Laucks et al (2000) found SD values of 11–30%, Sabban and van Hout (2011) found 16–20% and Hirose and Osada (2016) provided the lowest variation of 5–12%. Based on these and some further findings, several studies assumed an overall range of pollen grain density of 1.0–2.0 g/cm^3^ (Pope 2010; Griffiths et al. 2012) or 0.5–2.0 g/cm^3^ (Chen et al. 2019). Our results ranged from 0.595 g/cm^3^ (*P. sylvestris* aged) to 2.279 g/cm^3^ (*L. perenne* aged), while the results from our literature research reached from 0.167 g/cm^3^ (*Picea excelsa*, Jackson and Lyford 1999) to 2.274 g/cm^3^ (*Robinia pseudoacacia*, Jackson and Lyford 1999). The very low density found for *P. excelsa*, even if compared to other gymnosperm species, is surprising and probably should be validated once more by further experiments, before defining the lower limit of possible grain density. On the other hand, the so far collected results indicate that 2.3 g/cm^3^ is a good value for the upper limit of grains, which was indeed reached by some species in all groups of examined land plants (Fig. 12C).

The density of a pollen grain depends on the proportions of dry mass, water and air in the total mass and volume. It is commonly known that lignified plant cell wall has a density of 1.5 g/cm^3^. Similar values have been also reported for starch (Lam and Newton 1992). Thus, the dry mass of pollen can be assumed to have always a density above 1 g/cm^3^. The changes in water content and the potential air volume define the possible range of density for the grain. In general, a density clearly above 1 g/cm^3^ indicates a high percentage of dry mass and probably no air inside the grain. A density close to 1 g/cm^3^ indicates a high percentage of water and maybe some air pockets, and a density clearly below 1 g/cm^3^, like in gymnosperms, indicates a high percentage of air. Furthermore, with no air in a grain, its density should increase during drying, as the proportion of dry mass to water increases. This was also the result for *A. vulgaris*, *P. lanceolata* and *L. perenne* in our study, based on our assumption that there is no air inside the grains. Thus, we calculated the equivalent volume for the grain mass that we measured. The actual volume observed with microscopy might be larger and the actual density lower, if additional air is present within the pollen. However, with the strong variation in shape, observed for *P. lanceolata* and *L. perenne* and no variation in *A. vulgaris* (Fig. 1), we assume that the changes in mass and volume are indeed equivalent and that the effect of air is neglectable in these species. On the other hand, we observed a decrease of density in *P. sylvestris* and *C. avellana* during aging, indicating some additional air inside the aged grains. However, as we made no statistical analysis, we cannot tell whether the observed changes in density are significant. Considering the general uncertainty of pollen density calculation, it might in fact be only natural variation.

The pollen grains of *P. sylvestris*, like in many gymnosperm species, carry sacci with a constant air volume independently of harmomegathy (Fig. 1), resulting in their general low density. This is also a widespread adaptation in gymnosperms to a complex pollination mechanism, which requires ascension within a so-called pollination droplet (Owens et al. 1998). Thus, in this case, the amount of air in the pollen grains is functional and needs to be considered for density calculation. One study made 3D reconstructions and accurate volume measurements of fixated pollen grains from *Picea wilsonii*, based on ultrathin sections using so-called AutoCUTS-SEM (Shen et al. 2020). They provided a volume of 121,267 µm^3^ for the corpus and a volume of 26,659.3 and 26,393.3 µm^3^, respectively, for the two sacci, resulting in a total volume of 174,319.6 µm^3^. Thus, the percentage of air was around 30% of the total volume. While the state of the grain was not clearly defined, the shape provided in the images resembles a hydrated grain, meaning that in a dehydrated grain the percentage of air would be even higher. Therefore, this fits with the low density values that we found for *P. sylvestris* and other Pinaceae species. Interestingly, we made a spontaneous estimation of the volume of *P. wilsonii*, using the scale bar provided in the images in Shen et al. (2020) and the formula for two radii. This provided a small radius of 31 µm and a large radius of 45 µm, resulting in a calculated volume of 181,144,2 µm^3^, which was only 4% larger than the actual volume provided by Shen et al. (2020). This indicates that the two-radii approach can provide a very good approximation for the pollen grain volume, even in the case of irregular shaped grains like those of gymnosperms. However, more experimental comparisons with more precise methods for volume measuring are necessary to characterize the accuracy of the PPA approach.

### Comparison of literature results on eudicots, monocots and gymnosperms

The majority of the used mass, volume and density values from the literature was provided by the extensive review of Jackson and Lyford (1999), which themselves collected results from previous studies to characterize pollen settling velocity. In total, 46 of all 84 values were taken from the table in Appendix 2 of that study. We were not able to find all of the corresponding original studies they refer to. Therefore, we made no further validation of the provided data. However, we found that in 9 of 46 cases, all three parameters (mass, size and density) had been measured and in all 9 cases, there was a discrepancy between the density calculated from the provided mass and size information and the directly provided density value that was probably calculated from settling velocity measurements, using Stokes´s law (Suppl. Table S5). In six cases, this discrepancy was below 30%, which fits with the described overall uncertainty of density calculation. However, there was also one case with a discrepancy of more than 100% (*Plantago lanceolata*, Jackson and Lyford 1999). This indicates that in this case at least one of the three parameters was erroneous. Our own results for *P. lanceolata* fit much better with the provided density of 0.97 g/cm^3^, while the density calculated from mass and volume was 2.26 g/cm^3^. Thus, an error in the provided mass or volume seems more likely. Nevertheless, we included this value in our data sample, as it was still within the overall range. In general, measurements of settling velocity can also be affected for example by variation in pollen grain size and shape and have therefore been extensively discussed in previous studies (Jackson and Lyford 1999; Van Hout and Katz 2004; Sosnoskie et al. 2009). The study by Hirose and Osada (2016) also showed a discrepancy between the projected area equivalent diameters and aerodynamic diameters, calculated from settling velocity, for some pine pollen due to the effects of their sacci. In the same way, a discrepancy of density could also be caused by similar effects, in addition to errors in mass or volume.

Our evaluation of the provided literature data showed that it is reasonable to characterize eudicots, monocots and gymnosperms separately in terms of average pollen grain parameters. One needs to consider that this research is far from being complete and only provides an attempt for pollen grain characterization. Lots of further analysis on more species in randomly compiled samples are necessary to validate our findings and those of the literature. Nevertheless, some tendencies are visible. Among the examined species, gymnosperm pollen grains showed the largest distribution in size and mass, while their average density was significantly lower than that in angiosperms. On the other hand, monocots had a similar range of mass and size compared to small gymnosperms, despite having a larger average density, while eudicots were significantly smaller and lighter than all others, despite having a density similar to that of monocots.

The reconstruction of phylogenetic history of seed plants is to some degree highly associated with the observed changes in pollen morphology during evolution (Doyle 1978; Crane et al. 1995). In the early twentieth century, Wodehouse (1936) described three basic forms of pollen grains. The first type carries a triradiate crest and is common among Filicales and primitive gymnosperms, characterizing it as the probably most primitive form. The second one, the so-called monocolpate form, is characteristic for most gymnosperms, as well as monocots and basal dicots. The third type, the tricolpate form, characterizes the eudicots. Furthermore, the eudicots are recognized as a monophyletic group (Crane et al. 1995) and therefore clearly distinguished from the others in terms of the three basic forms. In this context, it is comprehensible that the size range of pollen from monocots and gymnosperms overlaps, while both can be distinguished from the pollen of eudicots.

It is also commonly known that the evolution of seed plants came along with a continuous reduction of the gametophyte (Qiu 2008), meaning also a reduction of pollen grain size. Our results showed no tendency for changes in average pollen grain density within the angiosperms. The observed variation showed that in individual species or certain groups within the angiosperms, the density might be significantly higher or lower than predicted by our regression analysis. Nevertheless, the average values appeared to be highly stable. Our original sample of literature reports for eudicots contained 18 values, resulting in an average density of 1.040 g/cm^3^, while for the final sample, containing 46 values, we found an average density of 1.060 g/cm^3^. Thus, the addition of 28 further values did not affect the average value at all.

Also splitting the sample in insect-pollinated and wind-pollinated species provided no significant difference. This also fits to the previous findings. Friedman and Barrett (2009) stated that the range of pollen size in wind-pollinated species is much smaller compared to insect-pollinated species, although their average size does not differ greatly. We can now assume that this is also true for pollen grain mass and density at least in the eudicots. However, especially the insect-pollinated species are vastly underrepresented in our sample: in future studies, their proportion compared to wind-pollinated species should be much higher. An extensive systematic analysis would be necessary to validate these results. In general, there are many natural factors that can influence pollen grain size (Muller 1979). The two major limits for anemophilous pollen grains are good air buoyancy, favoring smaller and lighter grains, on one hand, and capture probability by the stigma, favoring heavier grains on the other hand. The counteracting of both factors causes their small size range (Friedman and Barrett 2009). In this context, the large size variation in gymnosperms is surprising. The (equivalent) diameter in the provided literature values in our sample ranged from 20 µm to 100 µm, which is also the overall range that is stated for pollen of conifers in general. However, it is comprehensible that their low density provides good buoyancy, even for large grains and on the other hand, their capture mechanism with pollination droplets might not necessarily favor heavier grains (Owens et al. 1998), allowing a much larger variation in size. Again, our results must be treated with caution, due to the composition of the sample. Nevertheless, they indicate that species of the Cupressaceae family have small pollen, while species of the Pinaceae family have significantly larger pollen, except for the genus *Pinus*, which fits much better to the size of Cupressaceae pollen. Possible reasons for these findings might be addressed in further studies.

Considering the literature research for monocots, the Poaceae species also seem to be rather stable in terms of average density around a value of 1.168 g/cm^3^. Only our results for *L. perenne* show a large discrepancy to all other species (Fig. 8B). As mentioned before, the grain volume calculated from our results might be underestimated. The single value for *Butomus umbellatus* provided a remarkably lower density of 0.499 g/cm^3^. This indicates that the density variation among monocots might be higher than predicted by our regression analysis and a higher number of monocots species need to be examined, to test whether our findings need to be corrected or not.

Special attention must be given to the oversized grains of *G. hirsutum* and *Z. mays*. Mass and volume values of these two species were ten times larger than all other values in their respective plant groups, and in contrast to the gymnosperm sample, there were no grains with an intermediate size. As a consequence, the emphasis of these values on the regression line, if they were included, was much stronger than the combined effect of all other data points. Therefore, we chose to exclude them, as they are not representative for the average grain size. A comparison of regression analysis with and without these values is shown in Fig. S5 and S6 for eudicots and monocots, respectively. Muller (1979) stated that polyploidy is among the many factors that influence pollen grain size, with a higher level of polyploidy directly resulting in a larger grain. They further stated that from an evolutionary perspective, the effect of polyploidy is rather short-lived and easily overridden, but is still noticeable at an intraspecific level. The two species *G. hirsutum* and *Z. mays* are important crop plants and have been extensively cultivated until today. Thus, many of their properties differ from the plants evolved due to the natural selection. In this context, it is comprehensible that also their pollen grain parameters do not fit to the other values in our sample. In general, the effect of cultivation is an additional factor that needs to be considered before the pollen grain properties of a species in question can be predicted using the literature data. As crop plants are of special importance for humanity, this topic will probably become more important in future studies.

## Conclusions and outlook

Our study characterizes the three intrinsic parameters, such as mass, volume and density of single pollen grains, based on the study of five plant species analyzed in own experiments, as well as on an extensive literature review. We further highlight the importance of these parameters for the analysis of pollen grain mechanical properties in the provided example of our previous centrifugation experiment, as well as several references to previous studies. We also discuss some analysis methods used in previous studies, namely the weigh-and-count method and the projected-particle-area method. In spite of numerous factors and methodological challenges that may alter the results, we provide an average density of 1.06 g/cm^3^ for pollen of eudicots, with no difference between insect-pollinated or wind-pollinated species. We provide a density of 1.168 g/cm^3^ for Poaceae pollen that might be representative for monocots in general, but needs to be validated by analyzing more species in the future. Finally, we provide a significantly lower density of 0.422 g/cm^3^ for gymnosperms, namely conifers, that slightly differs between large Pinaceae pollen and small *Pinus* and Cupressaceae pollen.

While this research is far from being completed, these results for average density can be already used as a first approximation to estimate the mass of any single pollen grain for a given volume and the other way round, and can therefore contribute to further expand the current data banks of pollen grain properties. With this knowledge, the mechanical properties of pollen grains can be analyzed with relatively simple methods such as centrifugation (Huth et al. 2022; Becker and Gorb 2025), vibration (King and Buchmann 1996; Timerman et al. 2014) or drop test (Zafar et al. 2014), enabling vast quantitative data collection about pollen–surface interaction, adhesive properties and detachment conditions. This information can be further used in future studies to test hypotheses about how pollen grain properties such as mass, size, shape, surface structures and coating layers affect their adhesion, detachment, air buoyancy and dispersal and how these properties themselves are affected by changing environmental conditions due to climate change or pollution. Answering these questions is important, not only for complete understanding of the pollination process in general, but also for the behavior of pollen grains on various other substrates, for example in the context of allergenic pollen research or even artificial pollination.

## Supplementary Information

Below is the link to the electronic supplementary material.

Supplementary file1 (PPTX 52 kb)

Supplementary file2 (PPTX 53 kb)

Supplementary file3 (PPTX 51 kb)

Supplementary file4 (PPTX 73 kb)

Supplementary file5 (PPTX 653 kb)

Supplementary file6 (PPTX 645 kb)

Supplementary file7 (PPTX 953 kb)

Supplementary file8 (PPTX 8742 kb)

Supplementary file9 (DOCX 12 kb)

Supplementary file10 (DOCX 13 kb)

Supplementary file11 (XLSX 9 kb)

Supplementary file12 (XLSX 10 kb)

Supplementary file13 (XLSX 9 kb)

Supplementary file14 (XLSX 10 kb)

Supplementary file15 (XLSX 17 kb)

## Data Availability

Original data are provided as supplementary materials.

## Abbreviations

CI: 95% Confidence interval
ER: Equivalent radius
LM: Light microscopy
PPA: Projected particle area
SEM: Scanning electron microscopy
SD: Standard deviation
