# Supplementary material for: Characterizing the intrinsic parameters, mass, volume and density, of single pollen grains as a base for mechanical analysis of pollen adhesion
Source: Planta. 2026 Aug 7;264(3):87. doi: 10.1007/s00425-026-05111-x (PMC13451457; doi:10.1007/s00425-026-05111-x)
Supplement: Supplementary file 1 [file 425_2026_5111_MOESM10_ESM.docx]

**Supplementary Data S1**

**Detailed analysis of *C. avellana* centrifugation results**

In order to further understand the effect of pollen grain age on the measured adhesion force and to compare it to our previous results, we made a more detailed centrifugation analysis of *C. avellana* in a similar way as previously described for *A. vulgaris* (Becker and Gorb 2025). Therefore, the categories "aged” and “in contact” were further subdivided into “1 day”, “3 days” and “7 days”, resulting in 7 categories in total. For each category, we had 8 to 10 samples as simultaneous replications. The mean values of all sample replications per category were used for evaluation. We compared the percentage of detached grains in the round 1, round 2 and round 3, as well as the summarized percentage of detached grains in rounds 4-10 and the remaining grains after the round 10 between all categories. The results are shown in **Fig. 5**.

Following this evaluation, there was a remarkable difference between fresh and aged grains and also between fresh and in contact grains, while the difference between aged grains and in contact grains in general was mainly present at low rotation speed (round 1) and high speed (rounds 4-10 and remaining grains).

In detail, only 5.9 % of “fresh” grains got detached during the round 1, while the great majority got detached in the rounds 2 and 3, with 40.5 % and 30.1 %, respectively. Afterwards, 20.1 % got detached during rounds 4-10 and only 2.4 % remained attached after the round 10. In contrast, in the category "aged 1 day", already 58.2 % of grains got detached in the round 1. Thus, the percentage of detached grains in the following rounds was strongly reduced, compared to fresh pollen. This was less pronounced for the round 2 with 31.0 %, but more pronounced for the round 3 with only 7.7 % as well as for rounds 4-10 with 2.6 % and the remaining grains with only 0.5 %. After three days of aging, the percentage of detached grains in the round 1 was reduced again to 43.2 %, for the benefit of the round 3 with 16.1 %, while the percentage of detached grains in the round 2 remained rather similar with 36.2 %. This was also the case for rounds 4-10 with 3.9 % and the remaining grains with 0.7 %. Finally, after seven days of aging, the percentage of detached grains in the round 1 was further reduced to only 14.5 %, while slightly more grains got detached in the rounds 2 (46.4 %) and 3 (21.5 %).

Meanwhile, the percentage of detached grains in rounds 4-10 increased again to 16.4 %, which was comparable to fresh grains, and the percentage of remaining grains was low with only 1.3 %.

For the category “in contact 1 day”, we found a similar tendency as for corresponding aged grains, but less pronounced. Compared to fresh grains, the percentage of detached grains in the round 1 increased to 44.6 %. The percentage of detached grains in rounds 2 and 3 was reduced, compared to fresh grains, but very similar to “aged 1 day” grains with 32.2 % and 11.8 %, respectively. Furthermore, the percentage of detached grains in rounds 4-10 was also reduced, compared to fresh ones, but still higher than that of “aged 1 day” grains with 9.7 %, and the percentage of remaining grains was very low with only 1.6 %. After being in contact with the glass substrate for three days, the percentage of detached grains in the round 1 was reduced to 22.8 %, which was much less than for corresponding “aged 3 days” grains. This reduction was mainly for the benefit of detachment in rounds 4-10 which increased to 20.6 % again, while detachment in rounds 2 and 3 remained rather similar and comparable to “aged 3 days” grains, with 35.7 % and 19.0 %, respectively. Again, the percentage of remaining grains was low with only 1.9 %. Finally, after seven days in contact, the percentage of detached grains in the round 1 slightly increased again to 24.8 %, in contrast to “aged 7 days” grains. The percentage of detached grains in the round 2 slightly decreased and was much smaller than for “aged 7 days” grains, with 28.4 %, while the detachment in the round 3 remained the same and was very similar to “aged 7 days”, with 21.0 %. Also, the detachment in rounds 4-10 was comparable to “aged 7 days” grains, with 17.1 %, while the percentage of remaining grains showed the strongest difference of all, with an increase to 8.8 %. Based on these results, one can conclude that the adhesion of *C. avellana* pollen grains decreased during the first day for both, the aged and the in contact grains and was lowest after 1 day. Afterwards the adhesion increased again, while this increase was stronger for pollen in contact than for aged ones.
