# Supplementary material for: Characterizing the intrinsic parameters, mass, volume and density, of single pollen grains as a base for mechanical analysis of pollen adhesion
Source: Planta. 2026 Aug 7;264(3):87. doi: 10.1007/s00425-026-05111-x (PMC13451457; doi:10.1007/s00425-026-05111-x)
Supplement: Supplementary file 2 [file 425_2026_5111_MOESM9_ESM.docx]

**Fig. S1** Evaluation of centrifugation experiments, comparing the distribution of adhesion safety factor for all species in the three categories fresh, aged, and aged in contact with the substrate, showing all data. Data is shown in **Suppl. Table S1** and **S2**. Abbreviations as described in **Fig. 3** and **4**

**Fig. S2** Evaluation of centrifugation experiments, comparing the distribution of adhesion force [nN] for all species in the three categories fresh, aged, and aged in contact with the substrate, showing all data. Data is shown in **Suppl. Table S1** and **S2**. Abbreviations as described in **Fig. 3** and **4**

**Fig. S3** Summary of mass and volume measurements for all species analyzed by our own, showing the correlation between single grain volume [µm³] (x-axis) and single grain mass [pg] (y-axis). Vertical error bars show 95 % CI of weight measurements and horizontal error bars show SD of volume calculation. Corresponding data is provided in **Suppl. Table S3**. Abbreviations as described in **Fig. 3**

**Fig. S4** Summary of all own results and literature data, comparing the distribution of single grain mass (**A**), volume (**B**), and density (**C**) between all provided samples, with *G. hirsutum* and *Z. mays* included (see **Fig. 6-11**). Corresponding data is provided in **Suppl. Table S3** and **S5.** Abbreviations as described in **Fig. 12**

**Fig. S5** Results of simple linear regression analysis (SLR) for pollen of eudicots, based only on literature data, showing the correlation between single grain volume [µm³] (x-axis) and single grain mass [pg] (y-axis). It provides the average grain density as slope. **A** Regression analysis for all data. The dashed line shows the results with *G. hirsutum* included and the dotted line shows the results without *G. hirsutum*. **B** Comparison of pollination syndromes. Blue data dots indicate insect-pollinated species and orange data dots indicate wind-pollinated species. Corresponding data is provided in **Suppl. Table S5**

**Fig. S6** Results of simple linear regression analysis (SLR) for pollen of monocots, based only on literature data, showing the correlation between single grain volume [µm³] (x-axis) and single grain mass [pg] (y-axis). It provides the average grain density as slope. The dashed line shows the results with *Zea mays* included and the dotted line shows the results without *Z. mays*. **A** Regression analysis for all data. Red data point marks *Butomus umbellatus* data. **B** Only Poaceae data without *B. umbellatus*. Corresponding data is provided in **Suppl. Table S5**

**Fig. S7** Results of simple linear regression analysis (SLR) for pollen of gymnosperms, based only on literature data, showing the correlation between single grain volume [µm³] (x-axis) and single grain mass [pg] (y-axis). It provides the average grain density as slope. **A** Regression analysis for all data. **B** Regression analysis for small pollen (yellow data dots) and large pollen (blue data dots) separately. **C** Close up on small pollen. Corresponding data is provided in **Suppl. Table S5**

**Fig. S8** Cryo-SEM images, showing the comparison of fresh (**A**) and completely hydrated grains (**B**) of *Artemisia vulgaris*
